# 

**Published:** 1889-04

**Authors:** 


CONTENTS

VOL. 36 NO. 4.

| | PAGE. |
|---|---|
| The Death Penalty | 73 |
| Plea for Prisoners | 74 |
| History of a Dewdrop | 76 |
| Prayer of Socrates | 79 |
| Treatment of Obesity | 80 |
| Then and Now | 81 |
| The Pasteur Institute | 82 |
| Dates | 83 |
| Consumption, from a Mechanical Standpoint | 83 |
| Celluloise | 85 |
| Wonderful Intelligence of Dog | 86 |
| A Soldier's Life Saved by a Dream | 87 |
| The Anæsthetic Revelation | 88 |
| The Use of Oil to Still the Waves | 90 |
| A Conversation with the Grand Lama | 91 |
| Book Notices | 03 |
| Household | 94 |
| Miscellaneous | 95 |
| Winsome Brownie | 96 |

INDEX TO ADVERTISEMENTS OF HALF A PAGE AND OVER.

| | PAGE. |
|---|---|
| Murdock's Liquid Food Co | I |
| Esoteric Publishing Co | II |
| Zylonite | II |
| Western Land Investors' Opportunity | IV |
| Lactopeptine | V |
| Hollow Suppositories | VI |
| Revised Premium List | VIII |
| Rio Chemical Co | X |
| The Herb Cure Co | XI |

